# Contained rupture of an aortic arch aneurysm in a patient with syphilitic aortitis. A case report

**DOI:** 10.1590/1677-5449.210160

**Published:** 2022-01-07

**Authors:** Victor Bilman, Luca Bertoglio, Germano Melissano, Roberto Chiesa

**Affiliations:** 1 “Vita – Salute” University, Scientific Institute H. San Raffaele, Milan, Italy.

**Keywords:** tertiary syphilis, aortic aneurysm, aortic rupture, endovascular technique, sífilis terciária, aneurisma aórtico, ruptura aórtica, técnica endovascular

## Abstract

Syphilitic aortitis is a rare complication of tertiary syphilis, which can lead to aortic aneurysm formation, aortic valvular insufficiency, and ostial coronary stenosis. Syphilis has re-emerged worldwide over recent decades and vascular surgeons should be aware of its cardiovascular manifestations. Atypical clinical presentation, such as hemoptysis and a computed tomography angiography pattern of a thicker aneurysmal wall with ulcer-like aneurysm projections, should raise suspicion of syphilitic aortic aneurysm. An early diagnosis and appropriate surgical and medical therapies significantly contribute to successful treatment and favorable prognosis. Herein is reported the case of an 82-year-old male patient, positive for syphilis infection, with impending aortic arch aneurysm rupture treated with a hybrid arch repair. After 7 months, the patient was brought to the emergency room in cardiac arrest. Unsuccessful cardiopulmonary resuscitation maneuvers were performed, and an autopsy showed cardiac tamponade due to rupture of the ascending aorta.

## Resumo

A aortite sifilítica é uma complicação rara da sífilis terciária, que pode levar a formação de aneurisma aórtico, insuficiência valvar aórtica e estenose ostial coronariana. Ao longo das últimas décadas, a sífilis ressurgiu ao redor do mundo, e os cirurgiões vasculares devem estar atentos às suas manifestações cardiovasculares. Apresentações clínicas atípicas, como a hemoptise, e um padrão na angiotomografia computadorizada de parede aneurismática mais grossa com projeções de aneurisma com aparência de úlcera devem levantar a suspeita de aneurisma sifilítico da aorta. O diagnóstico precoce e terapias cirúrgica e clínica adequadas contribuem significativamente para um tratamento bem-sucedido e prognóstico favorável. Relatamos o caso de um paciente do sexo masculino, de 82 anos, que apresentou infecção por sífilis com ruptura iminente de aneurisma do arco aórtico. Ele foi tratado com reparo híbrido do arco. Após 7 meses, o paciente deu entrada na emergência com parada cardiorrespiratória. As manobras de reanimação cardiopulmonar foram realizadas, mas não obtiveram sucesso, e a autópsia mostrou tamponamento cardíaco devido à ruptura da aorta ascendente.

## INTRODUCTION

Over the past decade, the Center for Disease Control and Prevention (CDC) has reported an overall increase in syphilis infection rates in the United States, not limited to urban populations. The same pattern has been observed worldwide.^1^^,^^2^ Up to 30% of people exposed to the bacterium *Treponema pallidum* who do not get proper treatment will develop serious late complications, including neurologic, ocular, cardiovascular, and congenital syphilis, known as tertiary syphilis. This phase can manifest 10 to 30 years after the initial infection.^3^

Cardiovascular complications of tertiary syphilis are extremely rare. The most significant manifestation is known as syphilitic aortitis, which can lead to thoracic aneurysm formation, aortic valve disease, and coronary artery ostial stenosis.^1^^-^4 The prognosis of patients with untreated syphilitic aortic aneurysms can be devastating, with 2-year mortality of almost 80%.^5^^,^^6^ Therefore, correct diagnosis and appropriate surgical and medical therapies are crucial. Herein is reported the case of an 82-year-old male patient, positive for syphilis infection, with an impending aortic arch aneurysm rupture, who was treated with a hybrid arch repair in an urgent setting.

## CASE REPORT

**Figure 1:**
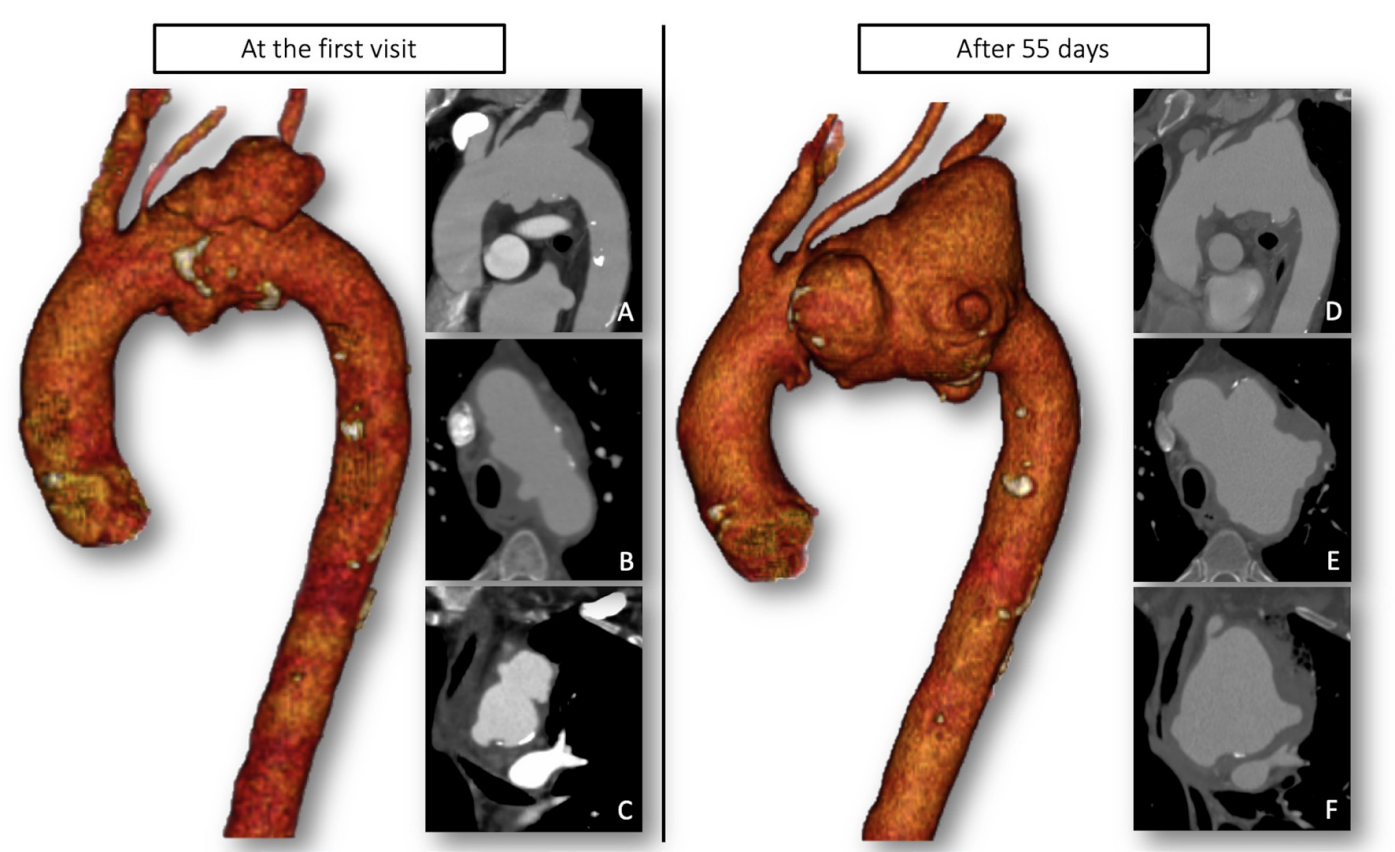
Preoperative computed tomography angiography (CTA) showing rapid growth of the syphilitic aortic arch aneurysm 55 days after the first visit to the outpatient’s clinic (A, B, C) and the urgent setting. (D, E, F) Sagittal (A/D), axial (B/E), and coronal (C/F) views of the two preoperative CTAs, almost two months apart.

An 82-year-old male patient with a past medical history of hypertension, mild chronic kidney injury, and chronic hepatitis C infection with liver cirrhosis was referred to our aortic outpatient clinic due to an arch aneurysm with a maximum diameter of 68mm (Figure 1) and a recent diagnosis of syphilis infection. The patient’s serum tests were positive for *Treponema pallidum* antibodies (by the T pallidum enzyme-linked immunosorbent assay method) and he was undergoing treatment with Benzathine penicillin. Other viral markers were negative. The patient was considered unfit for an open aortic arch replacement because of his comorbidities. A total endovascular aortic arch repair was proposed, and a custom-made stent-graft was ordered. At that time, a diagnosis of tertiary syphilis complicated by an aortic arch aneurysm was made.

**Figure 2:**
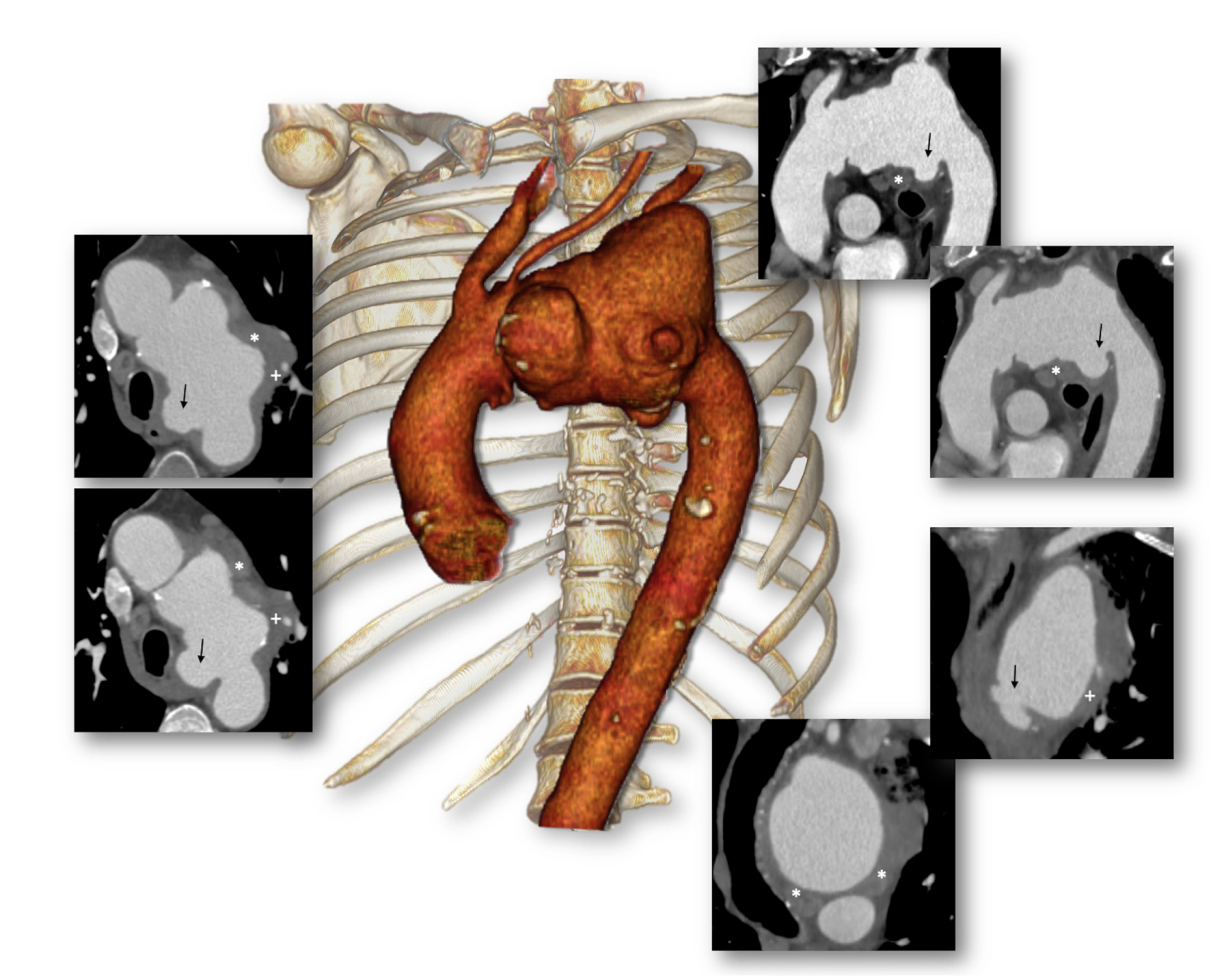
Preoperative CTA of the syphilitic aortic arch aneurysm characterized by focal and crescentic intramural hematomas (white *) - small regions of contrast accumulation with invisible or small communication with the true lumen, often with a peripheral connection with an intercostal or lumbar artery -, areas of eccentrically thickened aortic wall (white +), and ulcer-like projections with a wide neck (black arrows).

After 55 days, while waiting for the stent-graft to be manufactured, the patient came to the emergency department with hemoptysis, sudden chest pain, and a history of syncope. At admission, he was hemodynamically stable, with blood pressure of 110/70mmHg and a heart rate of 105 beats/min. Initial blood tests revealed a serum hemoglobin level of 8.9 g/dL, with no leukocytosis but C-reactive protein (CRP) of 221.1 mg/L. Cardiac complications were ruled out with a normal electrocardiogram (ECG) and no increase in troponin levels. Computed tomography angiography (CTA) showed the aortic arch aneurysm diameter had increased to 95mm from the origin of the brachiocephalic trunk to the upper descending aorta. (Figure 1) Signs of contained hemorrhage into the aortic wall from the vasa vasorum, aortic wall thickening, and multiple ulcer-like projections of the aortic lumen were identified (Figure 2). In this urgent setting, the patient was transferred to the operating room, and the vascular surgical team decided to perform a hybrid arch repair with debranching of supra-aortic vessels and thoracic endovascular aneurysm repair (TEVAR).

**Figure 3:**
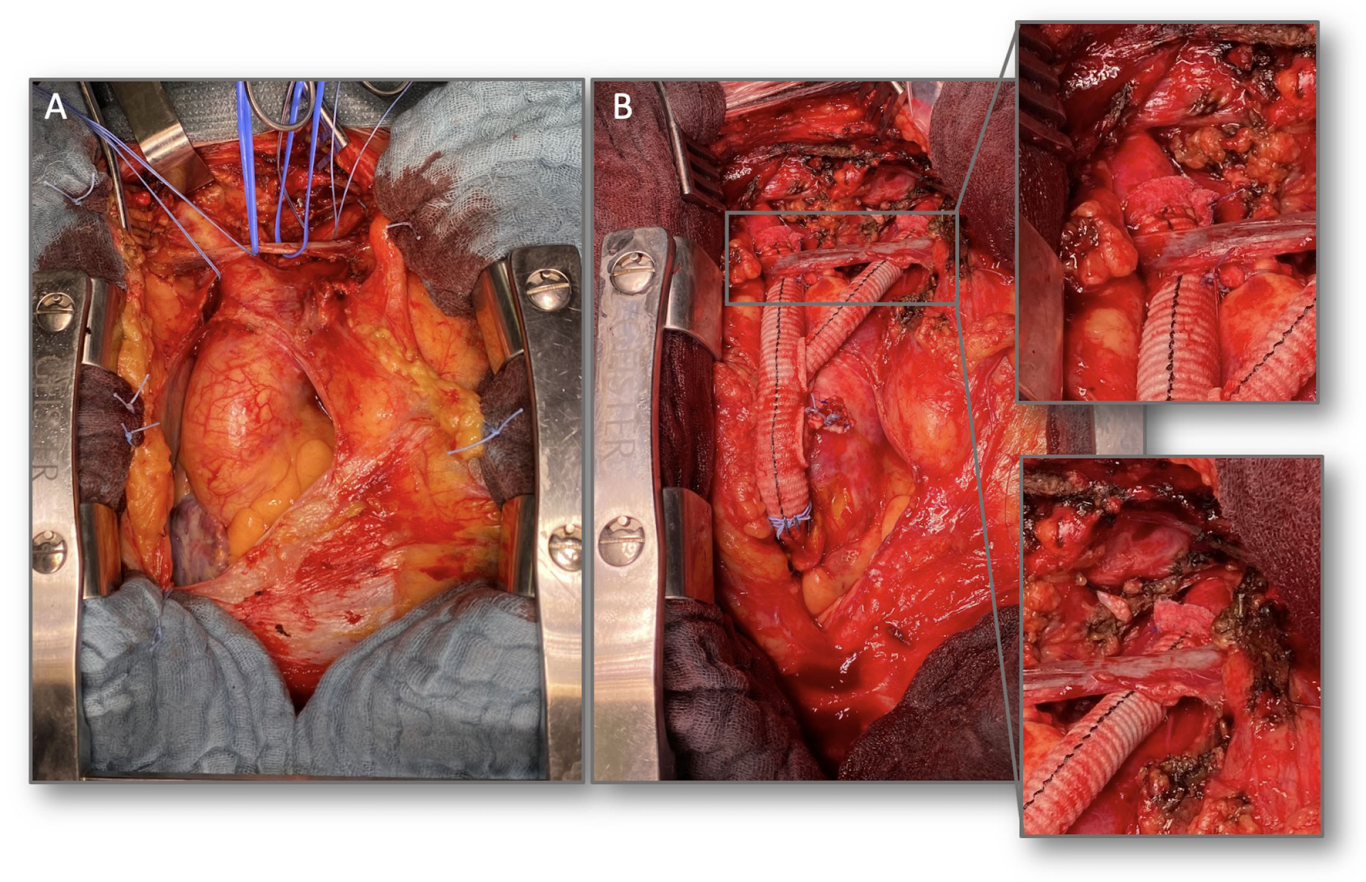
Intraoperative images. (A) exposure of the ascending aorta and the supra-aortic vessels (in this case: brachiocephalic trunk and left common carotid artery) through median sternotomy; (B) ascending aorta to the brachiocephalic trunk and left common carotid artery bypasses. Anastomoses to both vessels are highlighted. Note the ligature of the third branch of the trifurcated graft.

**Figure 4:**
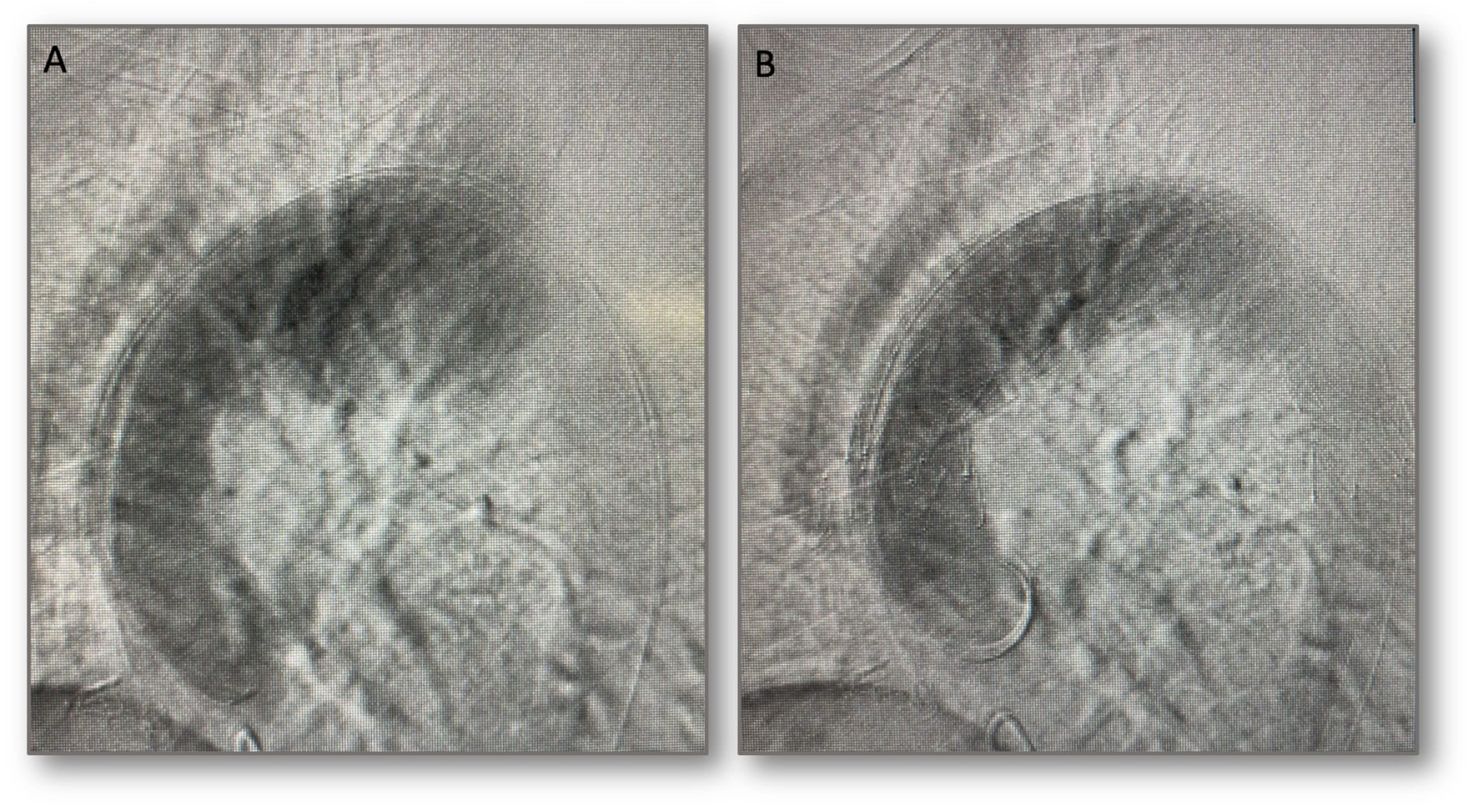
Endovascular component of the hybrid aortic arch repair. (A) pre-operative angiography showing patency of the supra-aortic vessels debranching’s and aneurysm perfusion; (B) after thoracic endovascular stent-graft repair, complete aneurysm exclusion was observed.

A median sternotomy was performed under general anesthesia and transesophageal echocardiography (TEE) monitoring, exposing the ascending aorta and arch vessels (Figure 3A). The proximal portion of a 12/8/8 x 300mm Hemashield trifurcated graft (Maquet, Rastatt, Germany) was anastomosed to the ascending aorta using a side-clamping technique. The 12mm distal branch was anastomosed to the brachiocephalic trunk (BCT) and the distal 8mm branch anastomosed to the left common carotid artery (LCCA) in an end-to-end fashion. Both BCT and LCCA were then ligated at their respective origins (Figure 3B). The left subclavian artery (LSA) was embolized using a 14 mm Amplatzer Vascular plug II (AVP II, St. Jude Medical, St. Paul, MN) through a left radial artery access, due to its highly posterior origin. Following this, through the right femoral artery and using rapid pacing, TEVAR was performed from zone 0 to the descending thoracic aorta using a Zenith Alpha thoracic stent graft (Cook Medical, Bloomington, Ind.). Control intraoperative angiography showed complete exclusion of the aneurysm (Figure 4).

**Figure 5:**
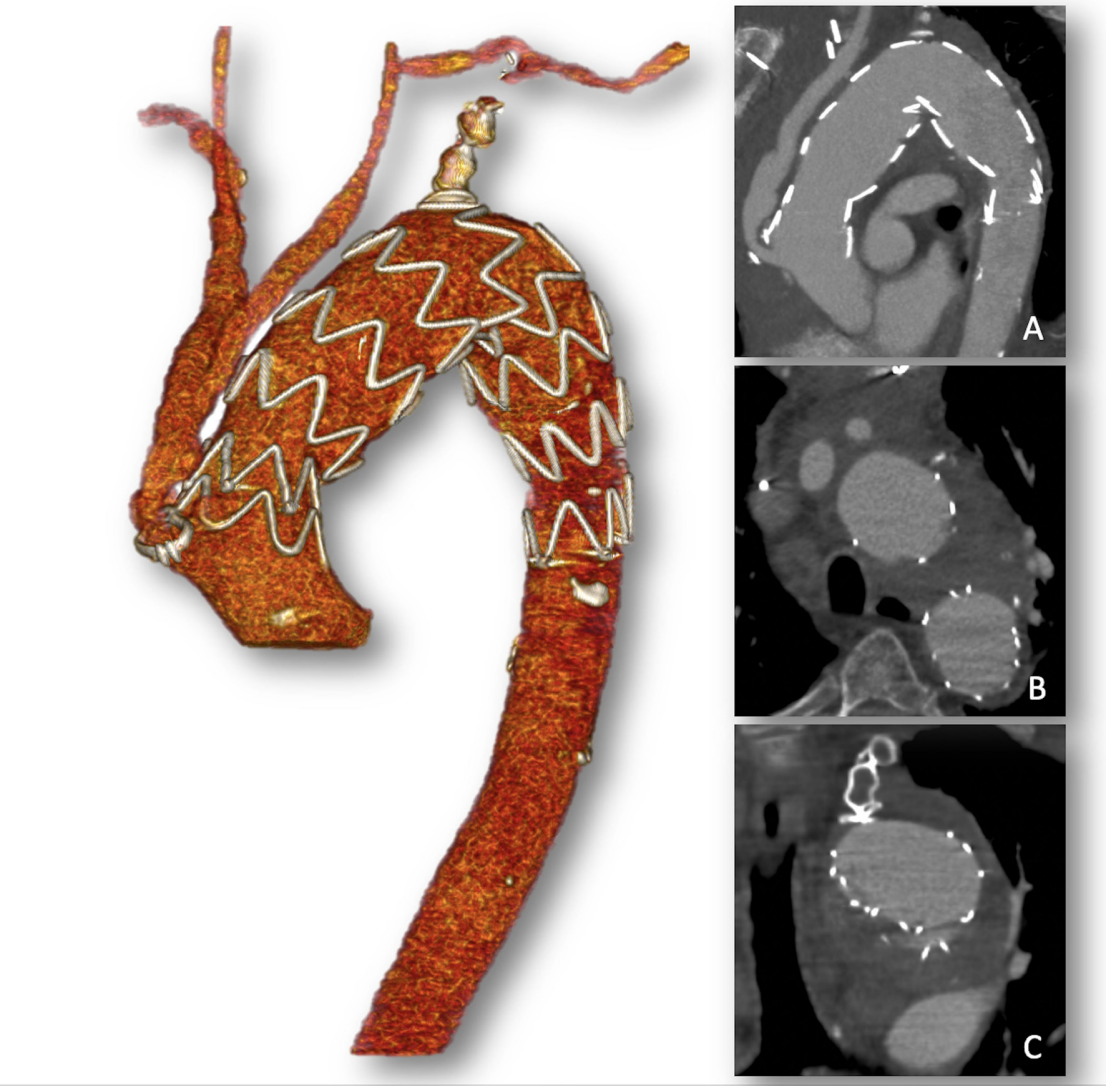
6-month follow-up computed tomography angiography 3D reconstruction showing complete exclusion of the aneurysm without any signs of endoleak, patency of the supra-aortic vessels, and no further aneurysm formation in the remnant aorta. (A) Sagittal view; (B) axial view; (C) coronal view.

The immediate postoperative day was uneventful, except for initial signs of left arm ischemia (pallor, pain, and paresthesia), for which it was decided to perform a left carotid-subclavian bypass using a 6mm Dacron graft. The patient remained hemodynamically stable in the postoperative period and was discharged on postoperative day 11. Six-month follow-up CTA showed complete exclusion of the aneurysm and no other aneurysm formation was observed in the remnant aorta (Figure 5). The following month, the patient was brought to the emergency room in cardiac arrest. Unsuccessful cardiopulmonary resuscitation maneuvers were performed. An autopsy showed cardiac tamponade due to rupture of the ascending aorta at the site of the debranching graft anastomosis.

This manuscript is in compliance with the Helsinki Declaration and with local ethical guidelines. The patient’s relatives consented to publication of this report.

## DISCUSSION

Recognition of syphilitic aortitis in the case of aortic aneurysms is crucial for better patient prognosis, since they would probably benefit from antibiotic therapy, mainly to prevent the occurrence of other tertiary syphilis manifestations and aortic aneurysm progression.^7^^,^^8^ Roberts et al.^7^ report that serological tests for syphilis are often negative in patients with cardiovascular manifestations, making histological analysis mandatory in open aortic surgery. The same authors also emphasize that if a syphilitic etiology is not suspected and, consequently, not recognized, the patient will not benefit from antibiotic therapy.^7^^-^9 The present case illustrates that, although rare, aortic syphilis has indeed re-emerged and now constitutes a cause of aortic aneurysm that vascular surgeons must be aware of and so should maintain a suspicion of this etiology.

After the initial infection, *Treponema pallidum* is present in the aortic wall, initially in the adventitia, and then in lymphatic vessels. Since ascending and thoracic aorta are rich in lymphatics, spirochetes have tropism for these aortic segments.^10^^-^13 The tendency of spirochetes to dwell in the small vessels of the *vasa vasorum* leads to an adventitial chronic inflammation, mainly in the arterioles that perfuse the media layer.^6^ The immune system response involves infiltration of plasmacytes and lymphocytes into the aortic wall layers, creating a diffuse and fibrous thickening of the intima.^7^ In fact, the vasa vasorum undergoes a process of endarteritis obliterans and necrosis of the media, and the elastic tissue of the vessel is destroyed and replaced by scar tissue.^11^^,^^12^ This inflammatory process can continue for a long time after the initial infection, which can explain the cardiovascular manifestations of syphilis aortitis generally occurring 10 to 30 years after the original untreated infection.^2^^,^^7^^,^^14^ Indeed, Heggtveit^15^ reported that among patients with syphilitic aortitis, 50% of the aortic aneurysms developed in the ascending aorta and 35% in the aortic arch, followed by 15% in the descending aorta.^2^^,^^15^ Although some cases of syphilitic abdominal aortic aneurysms have been reported,^16^^,^^17^ involvement of the infrarenal aorta is extremely rare because there are no *vasa vasorum* in that aortic segment.^7^^-^9

Clinical presentations are often atypical and depend on the segment of the aorta involved. Patients can remain asymptomatic for many years. Moreover, hazardous neurological or circulatory symptoms can arise due to compression or erosion into adjacent structures, such as hemoptysis, cardiac tamponade, and superior vena cava syndrome.^2^^,^^5^ Clinical diagnosis can also be based on imaging techniques. Computed tomography angiography imaging often shows thicker aneurysmal wall, a pattern of saccular projections on a fusiform aneurysm, focal, crescentic, intramural hematomas, and often with a peripheral connection with an intercostal or lumbar artery.^5^ Yuan^2^ reported that a saccular-type syphilitic aortic aneurysm is a significant predictive factor for poor patient outcomes. All these clinical presentations and imaging patterns should raise suspicion of syphilitic aortic aneurysm.

In the present case, the patient exhibited rapid progression of the aneurysm diameter, with almost 30mm in aneurysm growth in less than two months and impending signs of aortic rupture. At the first visit, the treatment proposal was a total endovascular aortic arch repair with a custom-made stent-graft; however, there is a manufacturing time between 4 and 8 weeks, and a non-negligible risk of adverse events occurring during this lead time has been reported in atherosclerotic aneurysms.^18^^,^^19^ Some factors, like aneurysm diameter greater than 70mm, have been statistically considered as leading to high-risk of rupture during the manufacturing time. The present case shows that syphilitic etiology should also be considered in the indications for custom-made endograft repair. Transposition of supra-aortic vessels and TEVAR for syphilitic aneurysms might be an alternative in case of patients unfit for open surgery and in urgent situations. However, surgeons should be aware that the ascending aorta anastomosis is being performed in an aorta segment that may be affected by syphilitic aortitis. This may have been the reason for the rupture of the anastomosis in the case presented, leading to an unfavorable outcome.

## CONCLUSION

Syphilitic aortitis is once more one of the causes of thoracic aortic aneurysms and vascular surgeons should be aware of this diagnosis in order to choose adequate medical and surgical therapies. Patients with syphilitic aortic aneurysms are at high risk of rupture and death. In an urgent setting, a hybrid arch repair with supra-aortic vessel debranching and TEVAR may be feasible. However, the ascending aorta can also be affected by syphilitic aortitis, which may lead to a pseudoaneurysm or aortic rupture at the proximal anastomosis site. Close lifelong surveillance is necessary due to the possibility of disease progression.
